# Transcriptomic Analysis of *Vibrio parahaemolyticus* Reveals Different Virulence Gene Expression in Response to Benzyl Isothiocyanate

**DOI:** 10.3390/molecules24040761

**Published:** 2019-02-20

**Authors:** Jie Song, Hong-Man Hou, Hong-Yan Wu, Ke-Xin Li, Yan Wang, Qian-Qian Zhou, Gong-Liang Zhang

**Affiliations:** 1 School of Food Science and Technology, Dalian Polytechnic University, Dalian 116034, China; sj101521@163.com (J.S.); houhongman2011@hotmail.com (H.-M.H.); LiKexin0903@hotmail.com (K.-X.L.); 18804208629@163.com (Y.W.); 15940283178@163.com (Q.-Q.Z.); 2 Graduate School of Environmental and Life Science, Okayama University, Okayama 700-8530, Japan; wuhongyan1908@hotmail.com

**Keywords:** *Vibrio parahaemolyticus*, benzyl isothiocyanate, virulence, RNA sequencing, qRT-PCR

## Abstract

*Vibrio parahaemolyticus* isolated from seafood is a pathogenic microorganism that leads to several acute diseases that are harmful to our health and is frequently transmitted by food. Therefore, there is an urgent need for the control and suppression of this pathogen. In this paper, transcriptional analysis was used to determine the effect of treatment with benzyl isothiocyanate (BITC) extracted from cruciferous vegetables on *V. parahaemolyticus* and to elucidate the molecular mechanisms underlying the response to BITC. Treatment with BITC resulted in 332 differentially expressed genes, among which 137 genes were downregulated, while 195 genes were upregulated. Moreover, six differentially expressed genes (DEGs) in RNA sequencing studies were further verified by quantitative real-time polymerase chain reaction (qRT-PCR). Genes found to regulate virulence encoded an l-threonine 3-dehydrogenase, a GGDEF family protein, the outer membrane protein OmpV, a flagellum-specific adenosine triphosphate synthase, TolQ protein and VirK protein. Hence, the results allow us to speculate that BITC may be an effective control strategy for inhibiting microorganisms growing in foods.

## 1. Introduction

*V. parahaemolyticus* is a Gram-negative, highly motile, halophilic bacterium that is always naturally found in marine environments and commonly isolated from seafood, including fish, crop, shrimp, scallops and oysters [1] due to water-feeding activity. Among the most important food-borne pathogens, *V. parahaemolyticus* can lead to a variety of symptoms, such as headache, fever, nausea, vomiting and watery diarrhea, within 24 h of infection [2]. In a report, on the eastern coast of China *V. parahaemolyticus* has caused 41% of food-borne disease outbreaks [3]. Moreover, *V. parahaemolyticus* is one of most severe food-borne pathogens that can spectacularly increase the mortality rate of aquatic life, and seriously infect humans [4]. Therefore, it is necessary to develop a wide variety of control measures to suppress *V. parahaemolyticus*.

Antibiotics have been used in aquaculture for a long time, however, after prolonged use the antibiotics accumulate in the seafood and result in bacteria gaining antibiotic resistance, and a large amount of antibiotics can even induce aplastic anemia in humans [5]. To help solve these situations, other kinds of food additives are commonly used in food processing, production and packaging to maintain the quality of food and limit the growth of pathogens. Many studies have concentrated on seeking new alternatives to prevent and treat food-borne pathogens. A previous report demonstrated that essential oils (EOs) from ginger and mustard could inhibit the growth of *V. parahaemolyticus* at 5 °C storage [6]. Feng et al. used RNA sequencing to prove that aqueous ozone could inhibit almost all genes in *V. parahaemolyticus* to kill the bacteria [7]. Formed from the digestion of glucotropaeolin in cruciferous vegetables, benzyl isothiocyanate (BITC) displays inhibitory effects against cancer due to glucosinolates. BITC can release biologically active isothiocyanates (ITCs) that play a main role in reducing carcinogens [8]. Previous studies mainly focused on the antibacterial and antifungal effects of BITC [9,10]. Dufour et al. demonstrated that BITC had the antimicrobial activities against *V. parahaemolyticus* [10]. However, no research has been conducted on the effect of BITC on *V. parahaemolyticus* at the transcriptomic level.

Thermostable direct hemolysin (TDH) is recognized as a major virulence factor of *V. parahaemolyticus* in causing human gastrointestinal disorders [11]. Previous reports have shown that the tdh gene is strongly related to clinical strains [12]. Banu et al. found that essential oils from *Cinnamomum tamala* could mediate the virulence factors of *V. parahaemolyticus* by influencing its biofilm, polysaccharides, lipopolysaccharides, flagellum and cytotoxins [13]. Therefore, the genes encoded by these proteins mentioned above are important for studying virulence factors. An effective tool for investigating pathogenesis is transcriptome analysis, which has widely been used to investigate gene expression changes, and to identify pathways that may be affected. Natural compounds that are potentially antimicrobial can provide novel and meaningful methods for inhibiting the growth of pathogens.

Hence, the purpose of this paper was to determine the differential transcriptional expression of the virulence genes of *V. parahaemolyticus* treated with BITC by means of RNA sequencing and to verify the expressions of significantly regulated genes (Table 1) by quantitative real-time polymerase chain reaction (qRT-PCR).

## 2. Results

### 2.1. Antibacterial Tests

By measuring the diameter of the inhibition zone (mm) of different sulfur compounds, we selected BITC as the inhibitor because it had the strongest antibacterial effect, with a minimum inhibitory concentration (MIC) of 9.54 µmol/L.

### 2.2. Global Changes at Transcriptome Level

Transcriptome analysis is a powerful tool for revealing the molecular mechanisms underlying the response of pathogens to food additives. Illumina RNA deep sequencing (RNA sequencing) technology generated two transcriptomic databases to further understand the pathogenicity of *V. parahaemolyticus*. We obtained a total 9.81 Gb of sequencing data, including 70,210,670 raw reads and 65,318,934 clean reads, with all the data having an average error rate of less than 0.02%. After removing low-quality and adapter sequences, 11,409,316, 12,152,830, 11,803,196 and 12,650,370, 9,990,268, 12,204,690 clean reads were obtained for C_BITC and Q_BITC samples, respectively. The Q20 and Q30 percentages were higher than 96% and 92%, respectively. In the C_BITC and Q_BITC samples, the average GC percentages were 48.29%, 48.39%, 48.35% and 48.20%, 48.01%, 48.08%, respectively. An overview of the transcriptome assembly statistics is shown in Table 2.

**Figure 1:**
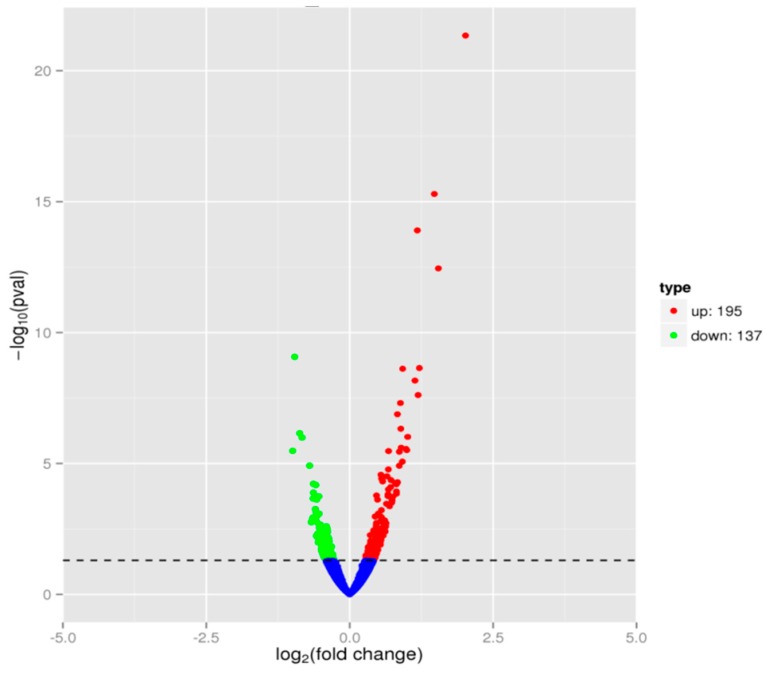
Volcano plot of differentially expressed genes (DEGs). In total, 332 genes present different expression levels. Red indicates upregulated expression, green indicates downregulated expression and blue indicates no significant differential expression when comparing Q_BITC with the C_BITC sample.

During *V. parahaemolyticus* growth in 1/4 MIC BITC in comparison to the sample without BITC treatment, gene expression levels were assessed by RNA sequencing technology to clarify molecular mechanisms of BITC on *V. parahaemolyticus*. To analyze and characterize the differentially expressed genes (DEGs) following BITC treatment, a *p*-value less than 0.05 was selected as the limitation for screening DEGs. A number of genes were found to be regulated under 1/4 MIC BITC treatment. In total, 332 genes presented a FPKM > 1 compared with the Q_BITC sample, implying that these genes were differentially expressed, of which 195 were upregulated and 137 were downregulated (Figure 1).

**Figure 2:**
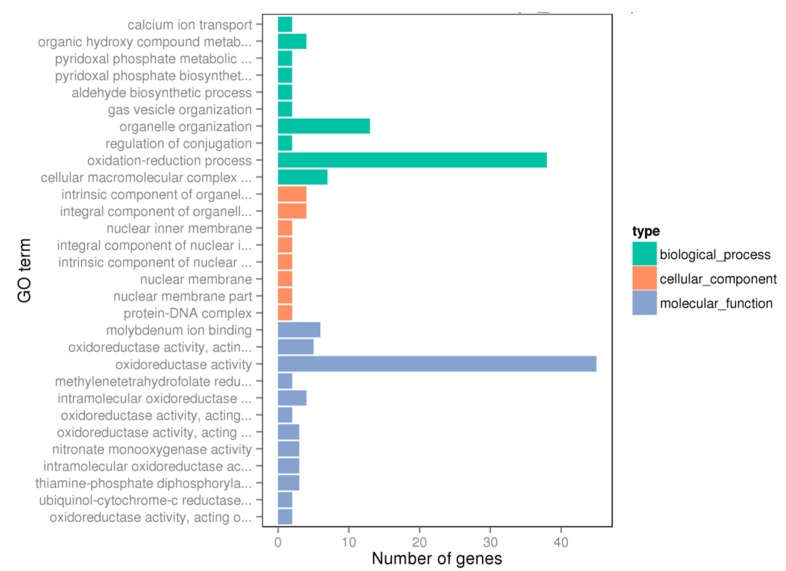
Functional enrichment of differentially expressed genes on gene ontology (GO) categorization. A total of 332 genes are assigned to 30 terms.

From the transcriptome data, gene ontology (GO) terms consisted of 332 unigenes. GO assignments are widely used to categorize gene functions, and the results regarding the effect of BITC on the expression of different genes in *V. parahaemolyticus* are displayed (Figure 2). Three domains, including biological process (10 subcategories), cellular component (8 subcategories) and molecular function (12 subcategories), comprised 30 major GO terms.

**Figure 3:**
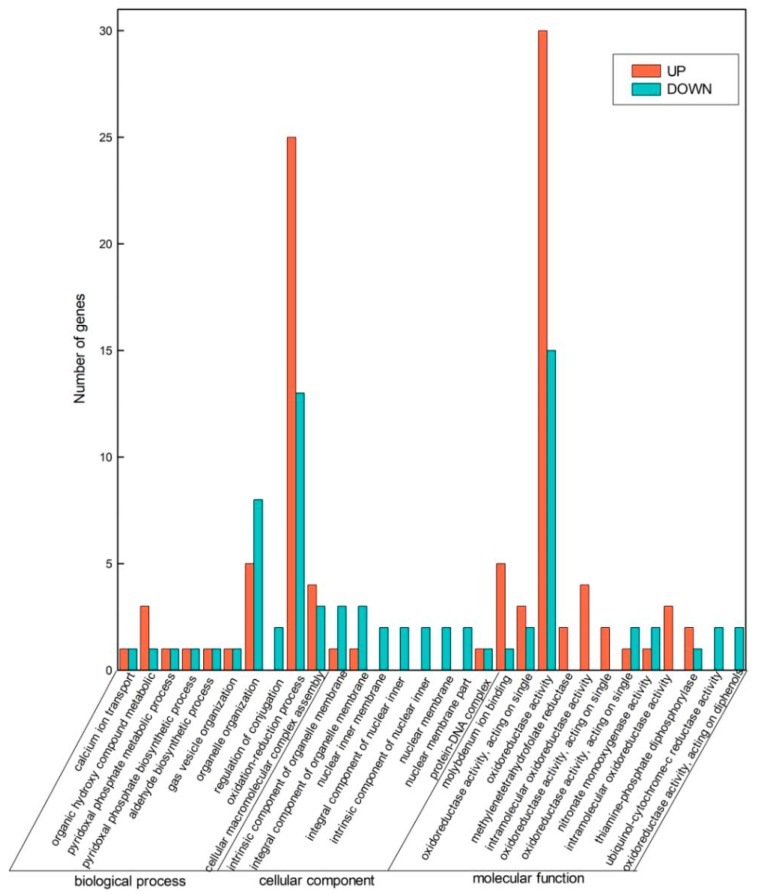
Transcriptomic analysis results. Differential expression of genes related to the functional categories biological process, cellular component, and molecular function of *V. parahaemolyticus* grown in the presence of benzyl isothiocyanate (BITC).

Significant changes in the biological process included organic hydroxy compound metabolic process, oxidation-reduction process, organelle organization, and cellular macromolecular complex assembly. The major changes in cellular component included an integral component of organelle membrane, intrinsic component of organelle membrane, nuclear inner membrane, and integral component of nuclear inner membrane. Molecular function mainly comprised molybdenum ion binding, acting on single donors with oxidoreductase activity, incorporation of molecular oxygen, oxidoreductase activity, and intramolecular oxidoreductase activity (Figure 3).

### 2.3. KEGG Pathway Analysis

**Figure 4:**
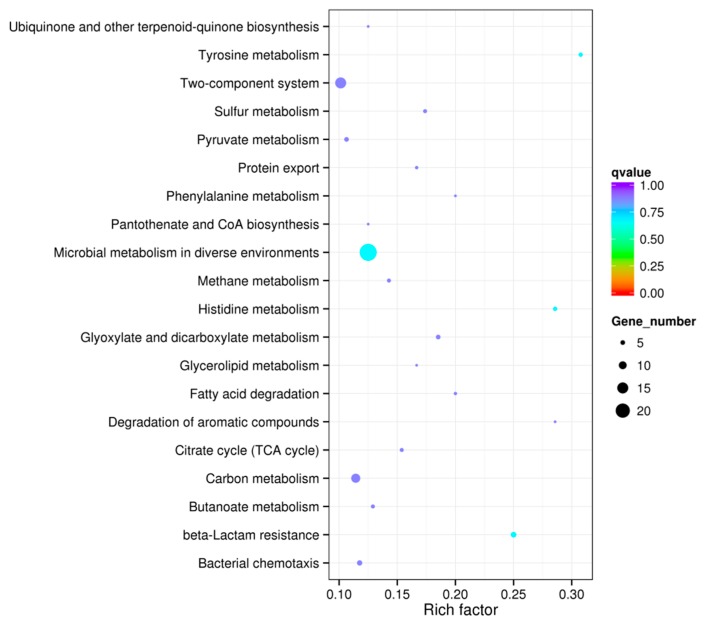
Kyoto Encyclopedia of Genes and Genomes (KEGG) classification of the differentially expressed genes. In total, 332 unigenes are assigned to 20 special KEGG pathways. Q value is an index used to determine the enrichment of the KEGG pathways. The closer the q value is to zero, the more significant the enrichment.

To identify the annotated unigenes-involved biological processes, Kyoto Encyclopedia of Genes and Genomes (KEGG) pathway analysis was carried out. In the KEGG analysis, DEGs were mapped to 65 reference pathways in KEGG database to analyze the functions of DEGs in the presence of BITC. DEGs with significant enrichment were mainly referred to 20 pathways (Figure 4), such as microbial metabolism in diverse environments, metabolic pathways, two-component systems, carbon metabolism, and biosynthesis of secondary metabolites. The results of KEGG pathway analysis revealed that bacteria treated with BITC displayed significantly enriched pathways.

### 2.4. Genes Related to Virulence and Validation the RNA Sequencing Data by qRT-PCR

First, qRT-PCR is a powerful tool to substantiate transcriptome data and analyze the gene expression level in *V. parahaemolyticus* to understand its virulence and the effect of BITC on its pathogenicity. Previous studies have already demonstrated the antimicrobial potential of BITC against various pathogens, such as *Escherichia coli* and *Campylobacter jejuni*. As mentioned above, it is similar to our findings in this study that BITC has antimicrobial potential against *V. parahaemolyticus*. Genes related to virulence, including *tdh*, *fliI*, *VPA0318*, *VP1057*, *VPA0243* and *VPA0202* were downregulated under BITC treatment in the RNA sequencing data as shown in Table 3. Therefore, the six genes mentioned above were selected for validation using qPCR.

**Figure 5:**
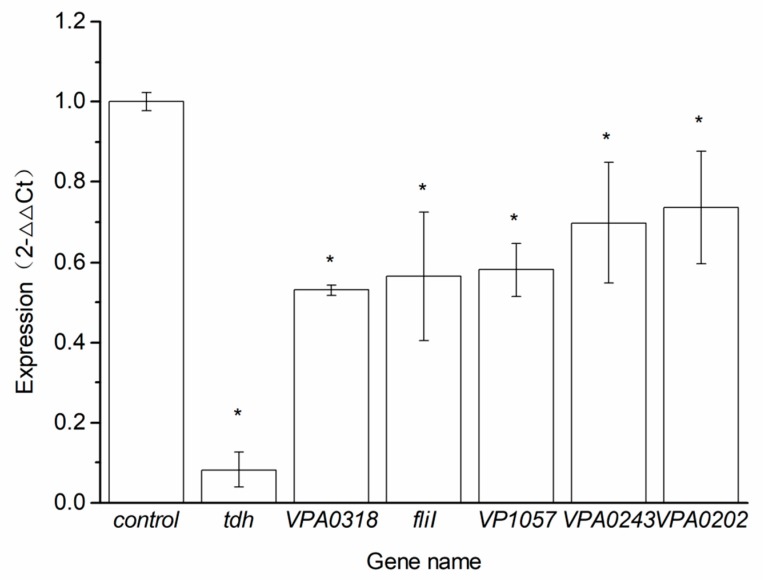
Validation of differentially expressed genes (DEGs) by qRT-PCR. Relative expression of *tdh*, *VPA0318*, *fliI*, *VP1057*, *VPA0243* and *VPA0202* compared with *16S rRNA* of the control normalized to one. The bar results are expressed as means ± SD from three independent replicates. * shows significant differences (*p* < 0.05).

The results of relative gene expression for *V. parahaemolyticus* treated with BITC are shown in Figure 5. From the histogram of these genes expression levels, it is clear that *tdh*, the most common virulence gene, was inhibited to the maximum extent as it dropped to 0.08 with BITC treatment. Additionally, the expression levels of *VPA0202*, *VPA0243*, *VP1057*, *fliI* and *VPA0318* were decreased compared with sample C_BITC. Significant downregulation in the expression of sample Q_BITC relative to sample C_BITC was exhibited in the qPCR results. Thus, the qPCR results were in accordance with our RNA sequencing data, thereby confirming those results.

## 3. Discussion

In regard to the antibacterial mechanism of natural products, the approach of transcriptomic analysis is useful to screen the differentially expressed genes in various pathogenic bacteria. Moreover, qRT-PCR is the direct method to validate the results of transcriptomic analysis. Therefore, several studies have combined transcriptomic analysis and qRT-PCR to explore the antibacterial mechanism in different pathogenic bacteria. Tan et al. have used RNA sequencing to screen six differentially expressed genes involved in biofilm formation of *Staphylococcus aureus* treated with ursolic acid, and further validated the gene expression level by qRT-PCR [14]. To explore the antibacterial mechanism of essential oil extracted from *Baccharis psiadioides* against *Listeria monocytogenes*, five differentially expressed genes associated with virulence were screened by RNA sequencing and confirmed by qRT-PCR [15]. Therefore, we investigated the antibacterial effect of BITC against *V. parahaemolyticus* at a transcriptomic level, and further studied the virulence gene expression in response to BITC treatment.

Seafood-borne illnesses and infections because of eating raw or undercooked oysters are caused by *V. parahaemolyticus* which is a human pathogen [16]. *V. parahaemolyticus* has led to severe losses in aquaculture in past years. Some available physical and chemical methods have been used to prevent and control *V. parahaemolyticus* infections in seafood [4,17]. Many kinds of food additives are widely used in the food industry to eliminate food-borne pathogens. For instance, adding grape seed extract to seawater can reduce the population of food-borne pathogens. Previous research has indicated the rapid and strong bactericidal effect of BITC on Gram-negative bacteria, while BITC can inhibit or may not affect the growth of Gram-positive bacteria [18]. Meanwhile, during the decontamination process, adding diverse amounts or concentrations will make a difference. With increasing global occurrences of *V. parahaemolyticus*, it is increasingly important to understand the virulence factors as well as the impacts on humans [17]. In this paper, we used BITC as a food additive to investigate the effect of BITC treatment on the gene expression response of *V. parahaemolyticus*. Virulence genes such as *tdh*, *VPA0318*, *fliI*, *VP1057*, *VPA0243* and *VPA0202* were downregulated (*p* < 0.05) in the presence of BITC. The major virulence factor of *V. parahaemolyticus*-TDH is coded by *tdh* [14], which is closely related to pathogenicity. Reported by an epidemiological study, TDH occupies an important position in leading severe pathogenic ability in *V. parahaemolyticus*, and it appears in almost all (95%) clinical isolates. TDH has the ability to lyse red blood cells and produce a special hemolysis ring on Wagatsuma blood agar plates [19]. ‘Kanagawa phenomenon’ is the phenomenon in which TDH is secreted and lyses red blood cells while simultaneously generating a hemolysis ring on Wagatsuma blood agar plates and is commonly related to gastroenteritis. Previous reports have also revealed that hemolytic activity and cytotoxicity of TDH are related to bacterial virulence.

Type III secretion systems (T3SSs) regulate the virulence of many animal and plant bacterial pathogens [20]. The peripheral membrane ATPase-FliI plays an important role in the type III protein export mechanism that depends on its bacterial flagellum [21]. Highly homologous to pathogenic bacteria type III secretion systems, FliI injects virulence effector proteins into the eukaryotic host cells for invasion [22]. The latest findings examined the flagellar adhesive and invasive properties, especially related to the flagellum are potential virulence factors [23].

From the transcriptome data, we found that the gene *VPA0243* encodes VirK protein, which is reported to be related to virulence in *Salmonella.* Typhimurium, and this study are the first to find VirK in *V. parahaemolyticus*. A previous report concluded that VirK was important in late stages of *Salmonella* enteric fever, and for the host environment, it most likely causes the remodelling of the bacterial outer membrane. The ∆*VPA0243* mutant of *S.* Typhimurium lessens virulence in a mouse infection model and decreases survival in macrophages [24]. In agreement with this, Spencer et al. demonstrated that *VPA0243* was the major virulence gene influencing survival and stays in the host for a long time [25]. VirK has a signal peptide for secretion through the Sec pathway into the periplasmic space [26]. VirK is recognized as a periplasmic protein that is necessary for the efficient secretion of plasmid-encoded toxins in *Escherichia coli* [27].

In many pathogenic bacteria, TolQ, one of the most widely distributed proteins, is one of the envelope proteins that constitutes the Tol system, contributing to colicin import and enhancing bacterial virulence. Abdelhamed et al. reported that the ∆*tolQ* mutant in *Edwardsiella ictaluri*, a Gram-negative pathogen, had reduced virulence in catfish compared to wild type, which suggested that the Tol system was beneficial for *Edwardsiella ictaluri* virulence [28]. In addition, Tol family genes are also important in the pathogenic Gram-negative bacteria such as *Haemophilus ducreyi*, *Vibrio cholerae* [29], *Salmonella enterica* [30], and *E. coli* [31]. Previous reports showed that TolQ, TolR, TolA and TolB were essential to *Salmonellae* for keeping the outer-membrane closed and contributing to its virulence [32,33]. Except for the GPL pathway, STm Tol-Pal proteins may influence other biochemical and metabolic mechanisms to promote the virulence of many pathogenic bacteria.

*VPA0202* encodes GGDEF domain, which plays a major role in synthesizing and degrading the nucleotide signal cyclic di-GMP (c-di-GMP) in some bacteria. A few years ago, it had been reported that c-di-GMP changed the pathogenicity of pathogenic bacteria and contributed to virulence. In *V. parahaemolyticus*, c-di-GMP-mediated regulation is balanced by regulating the ability of cell adhesion and movement, while changing the expression levels between the lateral flagellar and capsular polysaccharide genes [34]. A recent report on the mutant of *Pseudomonas aeruginosa* genes that encode GGDEF and/ or an EAL domain proteins suggested that intracellular c-di-GMP levels made a difference for the factors related to virulence, for example, through the type III secretion system to regulate cytotoxicity [35].

*VPA0318* encodes the outer membrane protein OmpV. Outer membrane proteins (OMPs) are widespread in Gram-negative bacteria [36] and are related to pathogenicity [37]. A previous study reported that outer membrane proteins could medicate the infection process including chemotaxis, motility through the mucous layer lining the intestinal wall, and survival of the gastric acid barrier [38]. Thus, outer membrane proteins are considered to be virulence factors.

## 4. Materials and Methods

### 4.1. Bacterial Strain and Growth Conditions

The experimental strain *V. parahaemolyticus* CGMCC 1.1614 was obtained from China General Microbiological Culture Collection Center. Frozen stocks of *V. parahaemolyticus* were streaked onto 3% sodium chloride tryptic soy agar (TSA-3% NaCl) and incubated at 37 °C overnight. A single colony was then transferred to 10 mL of 3% sodium chloride tryptic soy broth (TSB-3% NaCl) and cultured at 37 °C for 6 h to the logarithmic growth phase.

### 4.2. Antimicrobial Tests

First, to select the strongest antibacterial effect of sulfur, we chose four isothiocyanate compounds to initially understand their antibacterial ability: benzyl isothiocyanate (BITC), phenyl isothiocyanate (PITC), 3-(Methylthio) propyl isothiocyanate (MTPITC) and isoamyl isothiocyanate (IAITC). The antimicrobial activity of sulfur compounds was measured using the diffusion assay method. Twenty milliliters of sterilized TSA-3% NaCl was poured into a plate. After drying, the bacterical suspension was inoculated on the agar and mixed well. Afterwards, four filter papers were placed on the agar and 6 µl sulfur compounds were added to the middle of paper, leaving one paper as a control. Then, the plats were cultivated at 37 °C for 12 h. The diameter of the inhibition zone (mm) of different sulfur compounds was measured with a Vernier caliper.

### 4.3. RNA Extraction

*V. parahaemolyticus* was treated with BITC at concentration of 1/4 MIC (Q_BITC sample) or without BITC (C_BITC sample) as a control. Two samples of BITC were added in the logarithmic growth phase and shaken at 37 °C and 150 rpm for 6 h. Total RNA from the two samples (C_BITC, Q_BITC sample) was extracted separately using the RNAprep Pure Cell/Bacterial Kit (Tiangen Biotech, Beijing, China) following its manual. Total RNA was quantified by measuring OD260 nm/OD280 nm ratio. RNA contamination and degradation was confirmed on 1% agarose gels (*w*/*v*) stained with Goldview and visualized with a UV transilluminator (Versa Doc, Shanghai, China). All RNAs were stored at −80 °C immediately until use.

### 4.4. Library Preparation for Strand-Specific Transcriptome Sequencing

Three micrograms RNA was used for the RNA sample preparations. Sequencing libraries were produced using NEBNext^®^ Ultra™ Directional RNA Library Prep Kit for Illumina^®^ (NEB, Ipswich, MA, USA), and index codes were added to sequences. NEBNext First Strand Synthesis Reaction Buffer (5×) was used with divalent cations at a high temperature to obtain fragmentation. The first-strand cDNA was synthesized by M-MuLV Reverse Transcriptase (RNase H, NEB, Ipswich, MA, USA) and random hexamer primer (NEB, Ipswich, MA, USA). The second-trand cDNA was synthesized with RNase H and DNA Polymerase I. The remaining overhangs were converted into blunt ends via exonuclease/polymerase activities. The AMPure XP system (Beckman Coulter, Beverly, MA, USA) was used to screen cDNA fragments of 150~200 bp in length to purify the library fragments. Then 3 µL USER Enzyme (NEB, USA) was used with cDNA at 37 °C for 15 min followed by 5 min at 95 °C before PCR. Then, the reaction was carried out with Phusion High-Fidelity DNA polymerase kit (NEB, Ipswich, MA, USA), Universal PCR primers and Index (X) Primer. Finally, the Agilent Bioanalyzer 2100 system (G2939B, Agilent Technologies, Palo Alto, CA, USA) was used to assess products and library quality.

### 4.5. Bioinformatic Analysis

Clean data (clean reads) were acquired by removing reads including adapter, reads containing low quality reads and ploy-*N* from raw data. In the meantime, GC content, Q20 (percentage of bases with a Phred value > 20) and Q30 (percentage of bases with a Phred value > 30) were obtained. All downstream data were analyzed based on the clean data with high quality. FPKM, expected number of Fragments Per Kilobase of transcript sequence per Millions base pairs sequenced, as accounts for the influence of gene length for the read count and sequencing depth. Thus, it is the method for evaluating gene expression. Gene Ontology (GO) enrichment analysis of differentially expressed genes was performed using the GOseq R package, which can correct the gene length bias. GO terms with adjusted *p*-values below 0.05 were thought to be significantly enriched by differentially expressed genes. KEGG is a database resource for comprehending high-level functions and tools of the biological system from molecular-level information. We employed KOBAS software to verify the statistical enrichment of differentially expression genes in KEGG pathways.

### 4.6. qRT-PCR Validation of Differentially Expressed Genes

In order to verify differential gene expression, total RNA was treated with the PrimeScript™ RT Reagent kit with gDNA Eraser (TaKaRa, Dalian, China) to remove genomic DNA and reverse transcribed. SYBR^®^Premix Ex Taq™II (TliRNaseH Plus) (Takara, Dalian, China) was used for qRT-PCR following the manufacturer’s instructions. The *16S rRNA* gene was used as an endogenous gene and the differential gene expression level was assessed using the 2^−∆∆Ct^ method [39]. All reactions were carried out by using annealing temperature of 58 °C and a melting curve was obtained between 60 and 95 °C. All gene-specific primers were designed with Primer 5.0 software (PREMIER Biosoft, Palo Alto, CA, USA) and listed in Table 1.

### 4.7. Statistical Analysis

Data are shown as mean ± standard deviation (*n* = 3). Student’s t-test was used to assess the difference between two groups. Differences between each sample were recognized to be statistically significant at the *p* < 0.05 level.

## 5. Conclusions

The use of natural products can provide a new way to prevent and treat microorganisms in the food industry. This study is the first to report on the antimicrobial effect of BITC on *V. parahaemolyticus* at the gene level via transcriptome analysis, indicating a regulation of different virulence genes that leads to destabilization of bacteria. Further studies will focus on the screening of differentially expressed genes in a large scale. The protein level expression of the target genes as well as gene knock out studies should be performed to validate intricate regulations in biochemical and physiological properties of key targets.

## Figures and Tables

**Table 1:** Primers used to verify gene expression level by quantitative real-time polymerase chain reaction (qRT-PCR).

Gene	Primer	Sequence (5′→3′)
*16S rRNA*	16S rRNA-F	TATCCTTGTTTGCCAGCGAG
	16S rRNA-R	CTACGACGCACTTTTTGGGA
*tdh*	tdh-F	GGCATTTGGATGACCGAAGTA
	tdh-R	CTGACCAATCGCAACCACTTC
*VPA0318*	VPA0318-F	AGGTTACTTAGCGGGTGCG
	VPA0318-R	TTCACGGTCTTTGATGCC
*fliI*	fliI-F	TGCGGAACCCATCAACCC
	fliI-R	CGTCCGTCTTCGCCCAAA
*VP1057*	VP1057-F	CGGTTCAATCAGCCCATAC
	VP1057-R	AACGCTTCTGCGATACCTG
*VPA0243*	VPA0243-F	AACGCTTCTGCGATACCTG
	VPA0243-R	TTGCCATAGTGCGTCGTAGTCG
*VPA0202*	VPA0202-F	CGAAGAAGTGATGGTGGTG
	VPA0202-R	CTCGCATTGGTGAGTTGACG

**Table 2:** Summary of the RNA sequencing data.

Sample Name	Raw Reads	Clean Reads	Clean Bases (Gb)	Error (%)	Q20 (%)	Q30 (%)	GC (%)
C_BITC1	11,409,316	10,480,246	1.57	0.02	97.13	92.51	48.29
C_BITC2	12,152,830	11,176,478	1.68	0.02	96.93	92.11	48.39
C_BITC3	11,803,196	11,105,878	1.67	0.02	97.00	92.28	48.35
Q_BITC1	12,650,370	11,915,336	1.79	0.02	97.06	92.39	48.20
Q_BITC2	9,990,268	9,170,324	1.38	0.02	97.05	92.36	48.01
Q_BITC3	12,204,690	11,470,672	1.72	0.02	97.14	92.53	48.08

Q20: percentage of bases with a Phred value > 20; Q30: percentage of bases with a Phred value > 30.

**Table 3:** **Data** of the differentially expressed genes.

Gene_ID	Gene Name	log2 Fold Change (Q_BITC vs. C_BITC)	Pval (Q_BITC vs. C_BITC)	Padj (Q_BITC vs. C_BITC)	Significant (Q_BITC vs. C_BITC)
VPA1509	*tdh*	−0.32858	0.04469	0.62131	DOWN
VPA0318	-	−0.95983	8.44E−10	7.18E−07	DOWN
VP2246	*fliI*	−0.3946	0.029624	0.57503	DOWN
VP1057	-	−0.38624	0.049817	0.63785	DOWN
VPA0243	-	−0.49488	0.0045398	0.19316	DOWN
VPA0202	-	−0.4573	0.030909	0.58184	DOWN
